# Molecular basis of dimerization of lytic transglycosylase revealed by the crystal structure of MltA from *Acinetobacter baumannii*

**DOI:** 10.1107/S2052252521008666

**Published:** 2021-09-23

**Authors:** Hyunseok Jang, Hackwon Do, Chang Min Kim, Gi Eob Kim, Jun Hyuck Lee, Hyun Ho Park

**Affiliations:** a College of Pharmacy, Chung-Ang University, Seoul 06974, Republic of Korea; b Department of Global Innovative Drugs, Graduate School of Chung-Ang University, Seoul 06974, Republic of Korea; c Unit of Research for Practical Application, Korea Polar Research Institute, Incheon 21990, Republic of Korea; d Department of Polar Sciences, University of Science and Technology, Incheon 21990, Republic of Korea

**Keywords:** *Acinetobacter baumannii*, crystal structure, MltA, lytic transglycosylases, peptidoglycan remodeling, superbugs

## Abstract

Peptidoglycan digestion by murein-degrading enzymes is a critical process in bacterial cell growth and/or cell division. The membrane-bound lytic murein transglycosylase A (MltA) is a murein-degrading enzyme; it catalyzes the cleavage of the β-1,4-glycosidic linkage between *N*-acetylmuramic acid and *N*-acetylglucosamine in peptidoglycans. Although substrate recognition and cleavage by MltA have been examined by previous structural and mutagenesis studies, the overall mechanism of MltA in conjunction with other functionally related molecules on the outer membrane of bacterial cells for peptidoglycan degradation has remained elusive. In this study, the crystal structure of MltA from the virulent human pathogen *Acinetobacter baumannii* is characterized and presented. The study indicated that MltA from *A. baumannii* forms homodimers via an extra domain which is specific to this species. Furthermore, the working mechanism of MltA with various functionally related proteins on the bacterial outer membrane was modeled based on the structural and biochemical analysis.

## Abstract 2

MltA from the human pathogen *Acinetobacter baumannii* was characterized and its dimeric crystal structure was unveiled, demonstrating the extra-domain-mediated dimerization of MltA for the first time. Based on the current structural and biochemical studies, the working mechanism of MltA with various functionally related proteins on the bacterial outer membrane was modeled.

## 1. Introduction   

In bacteria, membrane-bound lytic murein transglycosylase A (MltA) catalyzes the exolytic cleavage of the β-1,4-glycosidic linkage between *N*-acetylmuramic acid and *N*-acetyl­glucosamine in peptidoglycan (Blackburn & Clarke, 2001 ▸; Lee *et al.*, 2013 ▸; Lommatzsch *et al.*, 1997 ▸). This murein-degrading enzyme-mediated peptidoglycan digestion is a critical process for the turnover and recycling of muropeptides during cell growth and/or cell division (Kraft *et al.*, 1999 ▸; Koraimann, 2003 ▸; Scheurwater *et al.*, 2008 ▸; Lee *et al.*, 2013 ▸, 2017 ▸). MltA belongs to the lytic transglycosylase (LT) enzyme family, a group of peptidoglycan-remodeling enzymes that are involved in the cleavage of glycosidic bonds of peptidoglycan in order to promote appropriate cell division and create spaces within the peptidoglycan to facilitate the assembly of membrane bacterial molecular machines, including flagella, pili and secretion systems (Scheurwater *et al.*, 2008 ▸; Lee *et al.*, 2013 ▸). A novel function of MltA as a growth factor that can support the growth and survival of *Francisella novicida* in arthropod species has been demonstrated by a recent study (Nakamura *et al.*, 2020 ▸).

**Figure 1:**
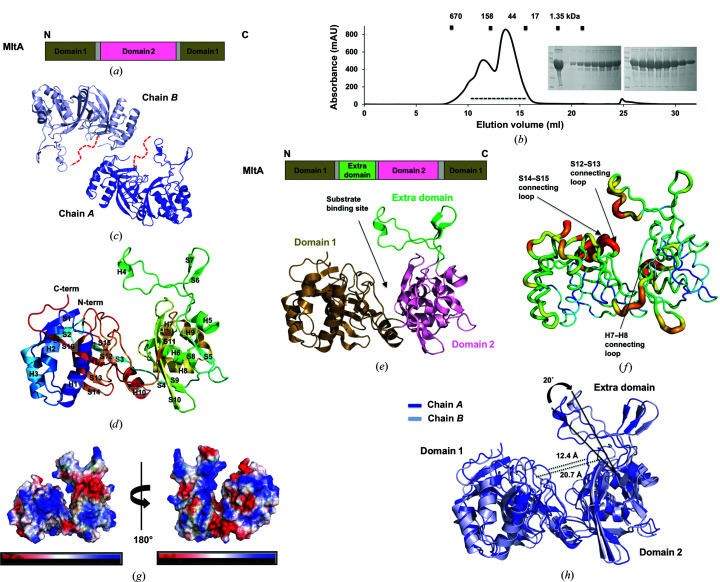
Crystal structure of abMltA. (*a*) General domain composition of MltA. (*b*) Size-exclusion chromatography profile. The results of SDS–PAGE performed to assess the identity and purity of the target protein are shown to the right of the main peak. The loaded fractions are indicated by a black dashed line. (*c*) Cartoon representation of two abMltA molecules in an asymmetric unit. Missing residues at the N-terminus of abMltA are shown by a red dashed line. (*d*) Rainbow-colored cartoon representation of monomeric abMltA. The chain is colored blue to red from the N-terminus to the C-terminus. Helices and sheets are labeled H and S, respectively. (*e*) A cartoon representation of the structure of abMltA showing the domain boundary in the structure. The putative substrate-binding site is indicated by a black arrow. (*f*) Putty representation showing the *B*-factor distribution in the structure of abMltA. Rainbow colors from red to violet in the order of *B*-factor values were used for visualization. Regions with the highest *B*-factor values are indicated by a black arrow. (*g*) Electrostatic surface of abMltA. (*h*) Superposition of the structures of the molecules in one asymmetric unit.

Based on the identification of different sets of consensus motifs, the structure of the catalytic fold and the domain organization, LT enzymes are classified into six unique families, and MltA belongs to LT family 2 (Dik *et al.*, 2017 ▸; Blackburn & Clarke, 2001 ▸; Alcorlo *et al.*, 2017 ▸). Sequence analysis revealed that MltA is unique among LT family 2. The typical consensus motifs involved in mediating the function of LT are not conserved in MltA (Blackburn & Clarke, 2001 ▸). In addition, a previous study showed that the structure of MltA is completely different from those of other members of the LT family (van Straaten *et al.*, 2007 ▸; Powell *et al.*, 2006 ▸; Jenkins *et al.*, 2019 ▸). MltAs from most species comprise two domains (domain 1 and domain 2). A deep groove is present between the two domains that can accommodate the glycan-strand substrate for cleavage [Fig. 1 ▸(*a*)] (van Straaten *et al.*, 2005 ▸). However, MltA from *Neisseria gonorrhoeae* contained an extra domain inserted in domain 2 (Powell *et al.*, 2006 ▸). Structural study of MltA complexed with a substrate analog revealed that the positions of domains 1 and 2 were shifted towards the bound substrate, with a narrower groove for enzyme reaction (van Straaten *et al.*, 2007 ▸). Studies of the enzyme activity of MltA from *Escherichia coli* indicated that Asp308 in the active site was critical for the activity of MltA as it served as a single general acid/base catalyst (van Straaten *et al.*, 2005 ▸; Barends *et al.*, 2005 ▸).

*Acinetobacter baumannii*, a typical rod-shaped Gram-negative bacterium, is a highly virulent human pathogen that causes hospital-derived infections; it is a notorious antibiotic-resistant pathogen that is considered to be a superbug. This bacterium is one of the ESKAPE (*Enterococcus faecium*, *Staphylococcus aureus*, *Klebsiella pneumoniae*, *A. baumannii*, *Pseudomonas aeruginosa* and *Enterobacter* spp.) pathogens currently on the top targets lists of the World Health Organ­ization and many pharmaceutical companies (Kumar, 2016 ▸; Burki, 2018 ▸). Being essential for cell growth and division, the LT enzyme family is considered to be an attractive target for antibacterial agents (Jenkins *et al.*, 2019 ▸; Lee *et al.*, 2013 ▸; Maitra *et al.*, 2019 ▸).

Although previous studies examining MltA from *E. coli* (ecMltA) have demonstrated the substrate-recognition and working mechanism of MltA (van Straaten *et al.*, 2007 ▸), the molecular basis underlying the oligomeric scaffolding assembly of MltA on the outer membrane with other functionally related molecules, such as MltA-interacting protein A (MipA) and penicillin-binding proteins (PBPs), which has been identified in several bacterial species, has remained unclear (Jenkins *et al.*, 2019 ▸; Vollmer *et al.*, 1999 ▸; Banzhaf *et al.*, 2020 ▸). In addition, the exact roles of the various LT families localized in the space between the two membranes of Gram-negative bacteria remain uncertain owing to the limited knowledge of their various substrates (Lee *et al.*, 2017 ▸). In this study, we characterize and present the crystal structure of MltA from the virulent human pathogen *A. baumannii* (hereafter called abMltA). Biochemical and structural studies revealed that abMltA forms homodimers in solution; these dimers may serve as the functional unit of this enzyme. The structure of abMltA showed a distinct extra domain in domain 2, which was similar to that in MltA from *N. gonorrhoeae*, and dimerization was mediated by this extra domain. Further, we propose the working mechanism of MltA with various functionally related proteins on the bacterial outer membrane based on the current structural and biochemical analysis.

## 2. Materials and methods   

### 2.1. Protein expression and purification   

The expression plasmid for N-terminally truncated MltA from *A. baumannii* corresponding to amino acids Gly23–Asn388 was constructed by inserting the synthesized gene product into a pET-28a vector. The gene sequence was derived from GenBank (ID SSM89718) and synthesized by BIONICS (Seoul, Republic of Korea). The expression plasmid was delivered into *E. coli* strain BL21 (DE3) using heat shock at 42°C. The transformed bacteria were spread on a lysogeny broth (LB) agar plate containing kanamycin and incubated for 16 h at 37°C. A single colony was picked and cultured overnight at 37°C in 5 ml LB containing 50 µg ml^−1^ kanamycin. Subsequently, the cells were transferred and cultured in 1 l LB. When the optical density, measured at 600 nm, reached approximately 0.6–0.7, the culture was cooled on ice and 1 m*M* isopropyl β-d-1-thiogalactopyranoside (IPTG) was added to the medium to induce gene expression. After adding IPTG, the cells were further cultured for 15 h at 20°C on a shaking incubator. Bacterial cells expressing the target gene product were harvested by centrifugation and the cell pellet was resuspended in 10 ml lysis buffer (20 m*M* Tris–HCl pH 8.0, 500 m*M* NaCl, 25 m*M* imidazole, 0.1 m*M* phenylmethanesulfonyl fluoride). The cells were disrupted in lysis buffer by sonication on ice with six bursts of 30 s each and a 60 s interval between each burst. To remove the cell debris and collect the supernatant, the cell lysate was centrifuged at 10 000*g* for 20 min at 4°C. The collected supernatant was mixed with nickel–nitrilotriacetic acid (Ni–NTA) resin solution (Qiagen, Hilden, Germany) by gentle agitation for 2 h at 4°C. The resulting mixture was loaded onto a gravity-flow column. The Ni–NTA resin was washed with 30 ml washing buffer (20 m*M* Tris–HCl pH 8.0, 500 m*M* NaCl, 60 m*M* imidazole) to remove unbound proteins. 2.6 ml elution buffer (20 m*M* Tris–HCl pH 8.0, 500 m*M* NaCl, 250 m*M* imidazole) was then loaded into the column to elute the bound protein. The resulting eluate was concentrated to 50 mg ml^−1^ and sequentially subjected to size-exclusion chromatography (SEC). SEC purification was performed using an ÄKTAexplorer system (GE Healthcare, Chicago, USA) equipped with a Superdex 200 Increase 10/300 GL 24 ml column (GE Healthcare) pre-equilibrated with SEC buffer (20 m*M* Tris–HCl pH 8.0, 150 m*M* NaCl). The peak fractions from SEC were pooled, concentrated to 33.8 mg ml^−1^, flash-frozen in liquid nitrogen and stored at −80°C until further use. Protein purity was assessed by SDS–PAGE.

### 2.2. Crystallization and data collection   

The high-quality crystal used for data collection was obtained from initial crystal screening using the hanging-drop vapor-diffusion method at 20°C. Initial screening was performed as follows: 1 µl 33.8 mg ml^−1^ protein solution in 20 m*M* Tris–HCl pH 8.0, 150 m*M* NaCl was mixed with an equal volume of reservoir solution and the droplet was allowed to equilibrate against 300 µl reservoir solution. The crystals were produced from a buffer comprising 0.1 *M* CHES pH 9.5, 20%(*w*/*v*) PEG 8000. They appeared in 60 days and grew to maximum dimensions of 0.2 × 0.2 × 0.4 mm. For data collection, the crystals were soaked in mother liquor supplemented with 30%(*v*/*v*) glycerol as a cryoprotectant solution, mounted and flash-cooled in a nitrogen stream at −178°C. The diffraction data were collected on the 5C beamline at Pohang Accelerator Laboratory (PAL), Pohang, Republic of Korea at a wavelength of 1.000 Å. The diffraction data were indexed, integrated and scaled using *HKL*-2000 (Otwinowski & Minor, 1997 ▸).

### 2.3. Determination and analysis of the structure   

Initial phase information for abMltA was obtained by molecular replacement with *MOLREP* (Vagin & Teplyakov, 2010 ▸) from the *CCP*4 suite (Winn *et al.*, 2011 ▸) using a partial model including part of domains 1 and 2 (residues 140–338) of the MltA structure from *N. gonorrhoeae* (PDB entry 2g6g; Powell *et al.*, 2006 ▸) as a search model. The initial model was constructed using *AutoBuild* in *Phenix* (Liebschner *et al.*, 2019 ▸). Further model building and refinement were performed using a combination of *REFMAC*5 (Murshudov *et al.*, 2011 ▸), *phenix.refine* (Liebschner *et al.*, 2019 ▸) and *WinCoot* (Emsley *et al.*, 2010 ▸). The quality of the model was validated using *MolProbity* (Chen *et al.*, 2010 ▸). Structural representations were generated using *PyMOL* (DeLano & Lam, 2005 ▸).

### 2.4. Multi-angle light-scattering (MALS) analysis   

The absolute molar mass of abMltA in solution was determined by MALS. The target protein, purified by affinity chromatography using Ni–NTA, was filtered with a 0.2 µm syringe filter and loaded onto a Superdex 200 10/300 gel-filtration column (GE Healthcare) that had been pre-equilibrated in SEC buffer (20 m*M* Tris–HCl pH 8.0, 150 m*M* NaCl). The mobile phase buffer flowed at a rate of 0.3 ml min^−1^ at 25°C. A DAWN TREOS MALS detector (Wyatt Technology, Santa Barbara, USA), which was interconnected with an ÄKTAexplorer system (GE Healthcare), was used for the MALS experiment. The molecular mass of bovine serum albumin was used as a reference value. The absolute molecular mass was assessed using *ASTRA* (Wyatt Technology).

### 2.5. Mutagenesis   

Site-directed mutagenesis was performed using a QuikChange kit (Stratagene, La Jolla, USA) according to the manufacturer’s protocols. Mutagenesis was then confirmed by sequencing. Mutant proteins were prepared using the method used for the purification of wild-type abMltA.

### 2.6. Sequence alignment   

The amino-acid sequences of abMltA from various species were analyzed with *Clustal Omega* (http://www.ebi.ac.uk/Tools/msa/clustalo/).

### 2.7. Accession codes   

Coordinates and structure factors have been deposited in the RCSB Protein Data Bank with PDB code 7esj.

## 3. Results and discussion   

### 3.1. Overall structure of abMltA   

The limited knowledge of MltA, with limited structural information, is often attributed to its insolubility due to its membrane-association. However, we found that a codon-optimized, signal peptide-removed version of abMltA (corresponding to amino acids Gly23–Asn388) was soluble. A rapid two-step chromatography, involving affinity chromatography followed by size-exclusion chromatography (SEC), generated a large amount of soluble abMltA protein (20 mg per litre of LB culture) with high purity (∼90% purity) [Fig. 1 ▸(*b*)]. During the SEC purification process, two homogeneous protein samples corresponding to oligomer and dimer fractions were produced, and only the dimer factions could successfully be crystallized [Fig. 1 ▸(*b*)]. The 2.06 Å resolution crystal structure of abMltA was finally resolved and refined to *R*_work_ = 21.77% and *R*_free_ = 26.40%. The crystallographic and refinement statistics are summarized in Table 1 ▸.

Two molecules, chain *A* and chain *B*, were found in the asymmetric unit [Fig. 1 ▸(*c*)]. The final model of each molecule was constructed from Ala44 to Asn388. 20 residues from the N-terminus could not be represented in the final model due to poor electron density that was not traceable. The crystal structure of abMltA showed the presence of ten α-helices and 16 β-sheets. It exhibited a typical domain composition, with two distinct domains: domain 1 and domain 2 [Fig. 1 ▸(*d*)]. Interestingly, however, abMltA contained an extra domain composed of residues Asp151–Asp184, which was distinctly separated from domain 2 [Fig. 1 ▸(*e*)]. Domain 1 consisted of four α-helices (H1–H3 and H10) and eight β-sheets (S1–S3 and S12–S16), while domain 2 consisted of five α-helices (H5–H9) and six β-sheets (S4–S5 and S8–S11) [Fig. 1 ▸(*d*)]. For the construction of domain 1, three α-helices (H1–H3) and three β-sheets (S1–S3) at the N-terminus formed a double-ψ β-barrel structure with one α-helix (H10) and five β-sheets (S12–S16) at the C-terminus, which is a common topology for MltA proteins [Figs. 1 ▸(*d*) and 1 ▸(*e*)]. Domain 2 was composed of ∼130 residues in the middle of MltA [Fig. 1 ▸(*e*)]. The extra domain, comprising one α-helix (H4) and two β-sheets (S6 and S7), was composed of ∼30 residues located between the N-terminal region of domain 1 and domain 2 [Fig. 1 ▸(*e*)]. *B*-factor analysis showed that three loops, the H7–H8 connecting loop in domain 2 and the S12–S13 connecting loop and the S14–S15 connecting loop in domain 1, demonstrated the highest *B*-factor values, indicating that these loops might be flexible [Fig. 1 ▸(*f*)]. All three loops with high *B*-factor values were located near the substrate-binding pocket. As demonstrated in a previous study performed with ecMltA, these loops were not directly involved in substrate interaction. These flexible loops around the active site might help to access the substrate in the active site of MltA (Powell *et al.*, 2006 ▸). Inspection of the surface charge distribution showed that negative and positive charged regions were evenly dispersed on the surface of abMltA [Fig. 1 ▸(*g*)]. A deep tentative substrate-binding pocket was distinctly formed between domain 1 and domain 2, including the extra domain [Fig. 1 ▸(*g*)].

The structures of the two chains in the same asymmetric unit were not identical, with a root-mean-square deviation (r.m.s.d.) of 3.2 Å [Fig. 1 ▸(*h*)]. Superimposition of chain *A* on chain *B* indicated that domain 2 and the extra domain were tilted 20° away from domain 1 when domain 1 was superimposed [Fig. 1 ▸(*h*)]. This domain rotation in chain *B* resulted in a wider substrate-binding pocket. The lengths of the entrance to the substrate-binding pocket in chain *A* and chain *B* were 12.4 and 20.7 Å, respectively. The interdomain movement induced upon binding of the substrate is one of the main characteristics of MltA (Powell *et al.*, 2006 ▸). Minimized interdomain movement, however, without substrate binding, which was detected in the current structure of abMltA, might be another interesting structural feature of MltA. This dynamic structural movement might be the optimal strategy for the recognition of a long substrate.

### 3.2. abMltA forms dimers in solution   

**Figure 2:**
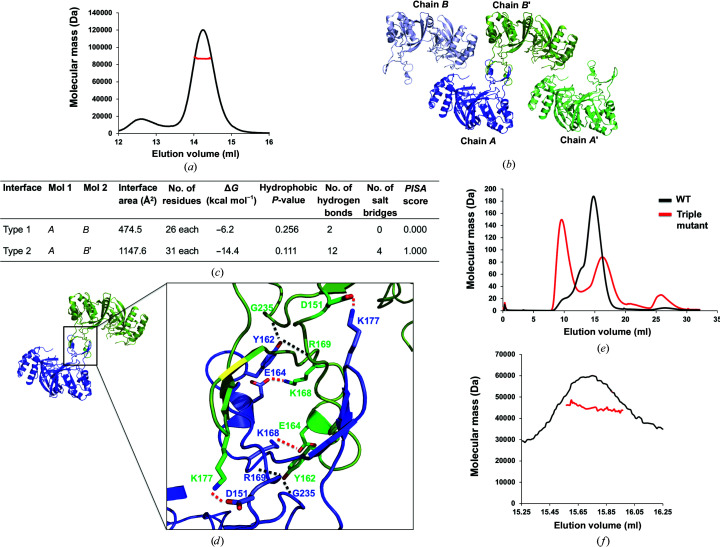
Dimeric structure of abMltA. (*a*) Multi-angle light-scattering (MALS) profile derived from the main size-exclusion chromatography (SEC) peak. The red line indicates the experimental molecular mass analyzed by MALS. (*b*) Crystallographic packing symmetry analysis. The two abMltA molecules found in the asymmetric unit are indicated by blue and light blue ribbon structures, while the other symmetric molecules are indicated by dark green and green ribbon structures. (*c*) Table summarizing the interaction details of the two types of interface analyzed by the *PISA* server. (*d*) Tentative dimeric structure of abMltA generated and analyzed by symmetry analysis and the *PISA* server. A magnified view of the region in the black box is provided to the right of the dimeric cartoon figure. Red and black dashed lines indicate salt bridges and hydrogen bonds, respectively. (*e*) Verification of the interfaces via mutagenesis. SEC profiles comparing the positions of eluted peaks between wild-type and mutant protein containing three mutated residues (Asp151 to Lys, Tyr162 to Trp and Glu164 to Lys). (*f*) MALS profiles of the triple mutant of abMltA. The red line indicates the experimental molecular mass analyzed by MALS.

Although its molecular architecture has not been determined by structural studies, a higher ordered murein-synthesizing machinery composed of PBPs, MipA and MltA has consistently been demonstrated by biochemical, biophysical and cellular studies (Vollmer *et al.*, 1999 ▸). As the stoichiometry of MltA in this machinery has not been determined, we analyzed the exact stoichiometry of abMltA in solution by calculating the absolute molecular mass using MALS. The main peak corresponded to an experimental molecular mass of 87.2 kDa (3.2% fitting error), which indicated a dimeric composition of abMltA, considering that the theoretically calculated molecular weight of abMltA is 42.4 kDa [Fig. 2 ▸(*a*)]. As we observed dimerization of abMltA in solution, we analyzed the crystallographic packing to search for symmetric molecules and to further understand the tentative dimeric structure of abMltA. An alternative dimeric structure was formed by chain *A* and chain *B*′, as demonstrated by crystallographic packing [Fig. 2 ▸(*b*)]. Since two types of interactions between chains *A* and *B* and between chains *A* and *B*′ can be formed for crystallographic packing, we analyzed the protein–protein interaction (PPI) in both the *AB* dimer and the *AB*′ dimer using the *PDBePISA* PPI-calculating server (Krissinel & Henrick, 2007 ▸). The interface of the *AB*′ dimer had a complex-formation significance score (CSS) of 1.000, where the score ranges from 0 to 1 as the relevance of the interface for complex formation increases, whereas the interface of the *AB* dimer has a score of 0.00 [Fig. 2 ▸(*c*)]. In the *AB*′ dimer the total dimer surface buried 2296 Å^2^ (a monomer surface area of 1148 Å^2^), which represents 6.7% of the total surface area. Hydrogen bonds (a total of 12) and salt bridges (a total of four) were the main forces responsible for the formation of this dimeric interface [Fig. 2 ▸(*c*)]. Asp151, Glu164, Lys168 and Lys177 from one molecule formed salt bridges with the same residues from the other molecule. Tyr162, Arg169 and Gly235 from each molecule were involved in the formation of hydrogen bonds at the dimeric interface [Fig. 2 ▸(*d*)]. In the case of the *AB* dimer, the dimer buried a total surface area of 918 Å^2^ (a monomer surface area of 474 Å^2^), which represents 2.8% of the total surface area as calculated by *PDBePISA*. This interface analysis indicated that the *AB*′ dimer might be formed in solution [Fig. 2 ▸(*c*)]. To confirm this result, we performed a mutagenesis study. Since Asp151, Tyr162 and Glu164 are the primary interface residues involved in the formation of the *AB*′ dimer, they were mutated to lysine, producing a D151K, Y162K and E164K triple mutant. We analyzed the effect of the mutations on the formation of dimers using SEC. Although the single mutants did not affect dimer formation (data not shown), as indicated in Fig. 2 ▸(*e*) the triple mutant (a protein with three mutations: D151K, Y162W and E164K) showed a definite disruptive effect, demonstrating a new peak corresponding to the size of a monomer in the SEC profile. The newly formed peak at the void volume might be attributed to aggregated triple mutant of abMltA [Fig. 2 ▸(*e*)]. This aggregated particle formed by mutation might be explained by (i) the direct effect of the triple mutation on the solubility of abMltA or (ii) the disruptive effect of the triple mutation on the formation of the dimer and the production of insoluble monomer. The molecular mass of the newly generated tentative monomer peak following triple mutagenesis was further calculated by MALS to confirm the mutagenesis effect. The MALS results showed that the molecular weight of the peak corresponding to the size of a monomer produced by the triple mutant was 44.8 kDa, indicating that this peak indeed represented the monomeric form of abMltA that was produced by disruption of the *AB*′ dimer interface. These results strongly indicated that abMltA forms a dimer in solution. The dimer formation was mediated by the extra domain of abMltA.

### 3.3. Comparison of the structure of abMltA with those of MltA from other species   

In a search for clues to infer the molecular mechanism of dimer formation and structural flexibility of each domain of MltA, we investigated its structural homologs using the *DALI* server (Holm & Sander, 1995 ▸). Although it exhibits critically important functional roles in the turnover and recycling of peptidoglycan during cell growth and cell division, the molecular structure of MltA from only two different species, *N. gonorrhoeae* (ngMltA) and *E. coli* (ecMltA), has been studied. Hence, these ortholog structures were selected as structural homologs from the *DALI* server. Considering that most bacteria contain an MltA enzyme, it is interesting that only two ortholog structures have been examined to date. This might be because MltA is localized on the membrane, making its structural study difficult to perform because of solubility issues. ngMltA (PDB entry 2g6g), the MltA domain of LtgG (PDB entry 6qk4) and two different states of ecMltA (open and closed; PDB entries 2pi8 and 2pic), in that order, were the top four matches obtained using the *DALI* server (Table 2 ▸).

**Figure 3:**
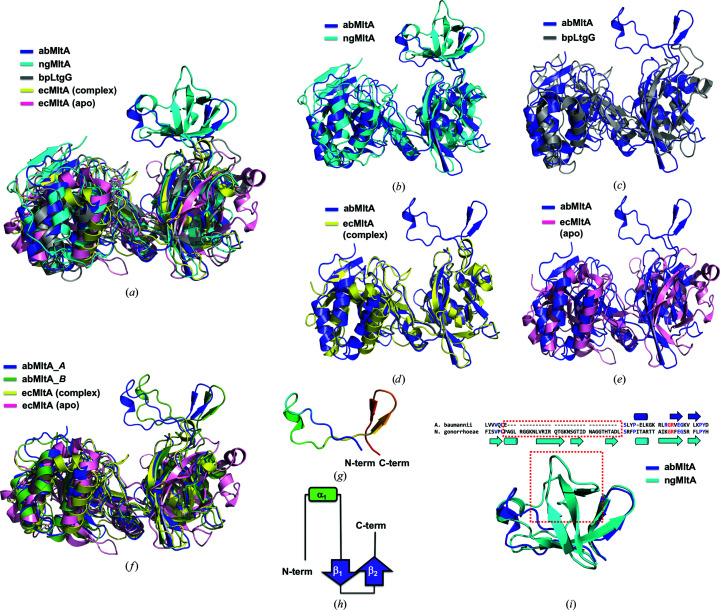
Structural comparison of abMltA with MltA enzymes from different species. (*a*) Structural comparison of abMltA with other orthologs. Currently available structures of MltAs from *N. gonorrhoeae* (ngMltA; PDB entry 2g6g), *B. pseudomallei* (bpMltA; PDB entry 6qk4) and *E. coli* (ecMltA; PDB entry 2pi8 for the complex structure with a substrate analog and PDB entry 2pic for the apo structure) were superimposed with the structure of abMltA. (*b*–*e*) Pairwise structural superimposition of abMltA with ngMltA (*b*), bpLtgG (*c*), ecMltA (complex structure) (*d*) and ecMltA (apo structure) (*e*). (*f*) Structural comparison of both chains of abMltA with closed and open forms of ecMltA demonstrated by superimposition. (*g*) The structure of the extra domain of abMltA. The chain is colored blue to red from the N-terminus to the C-terminus. (*h*) Topological representation of the extra domain of abMltA. N-term and C-term indicate the N-terminus and C-terminus, respectively. (*i*) Structural and sequence comparison of the extra domain of abMltA with that of ngMltA by superimposition. The red dotted box indicates the additional residues and structure in ngMltA. Secondary structures are presented with cylinders for α-helices and arrows for β-sheets. Completely conserved residues are colored red.

The structural comparison performed by superposition of monomeric abMltA with MltA from different species showed that the domain compositions and locations were different between each species, exhibiting an r.m.s.d. of 3.2 Å with ngMltA, 5.6 Å with LtgG, 2.7 Å with an ecMltA–substrate complex and 8.8 Å with ecMltA, although the overall folds of domain 1 and domain 2 were similar [Table 2 ▸ and Fig. 3 ▸(*a*)]. In particular, ngMltA, the protein with the highest structural similarity according to the *DALI* search, showed an additional loop and β-sheet in domain 1 [Fig. 3 ▸(*b*)]. Although the overall fold of domain 1 of ngMltA was quite different from that of abMltA, interestingly the extra domain of abMltA was located in the same position as that of ngMltA [Fig. 3 ▸(*b*)]. However, the structure of the extra domain of ngMltA differed from that of abMltA [Fig. 3 ▸(*b*)]. A pairwise structural comparison showed that the locations of domain 1 and domain 2 were not identical [Figs. 3 ▸(*c*), 3 ▸(*d*) and 3 ▸(*e*)]. The structural dynamic movement of each domain of MltA observed upon accommodating the substrate in ecMltA is one of the main features of MltA (van Straaten *et al.*, 2007 ▸). Substrate binding to ecMltA induced the reorientation of the two structural domains, domain 1 and domain 2, closing the deep groove of the active site, thereby resulting in a closed conformation [Fig. 3 ▸(*f*)]. The structure of chain *A* of abMltA was more similar to that of the closed conformation of ecMltA. However, abMltA did not include the substrate, indicating that substrate binding might not be the only reason for domain movement. Although domain 1 and the extra domain of chain *B* were shifted outwards a little compared with those of chain *A*, the structure of chain *B* was also more closely related to the closed conformation when compared with that of the open conformation of ecMltA [Fig. 3 ▸(*f*)].

Structural comparison studies indicated that the structure of ngMltA was most closely related to that of abMltA, along with the presence of an extra domain, indicating that the structure of abMltA most closely resembles the structure of ngMltA from an evolutionary perspective. The function of the extra domain has not been determined so far. Its structure in abMltA was simply composed of one α-helix and two β-sheets [Figs. 3 ▸(*g*) and 3 ▸(*h*)]. To speculate on the function of this extra domain by comparing the structural homologs, we again performed a *DALI* search (Holm & Sander, 1995 ▸). We failed to find any structural homologs, indicating that this fold was novel and had not been studied. Comparison of the structure of the extra domain of abMltA with that of ngMltA showed that ngMltA contained a structural motif similar to that of the extra domain of abMltA. However, the structure of the extra domain of ngMltA was more complex, containing four more β-sheets and one more α-helix in the middle of the domain [Fig. 3 ▸(*i*)]. About 35 additional residues in ngMltA formed a structurally different domain compared with that of abMltA. According to a previous structural study, the structure of ngMltA was solved in a monomeric form and the function of this extra domain has been suggested to be a protein–protein interaction module that might be used for binding to other proteins (Powell *et al.*, 2006 ▸). Although dimeric ngMltA was possibly lost by the authors, it might be possible that the more complicated extra domain detected in ngMltA is not responsible for dimerization. This structural variation of the extra domain might indicate the functional diversity of this domain.

### 3.4. Proposed mode of substrate recognition by abMltA on the outer membrane   

**Figure 4:**
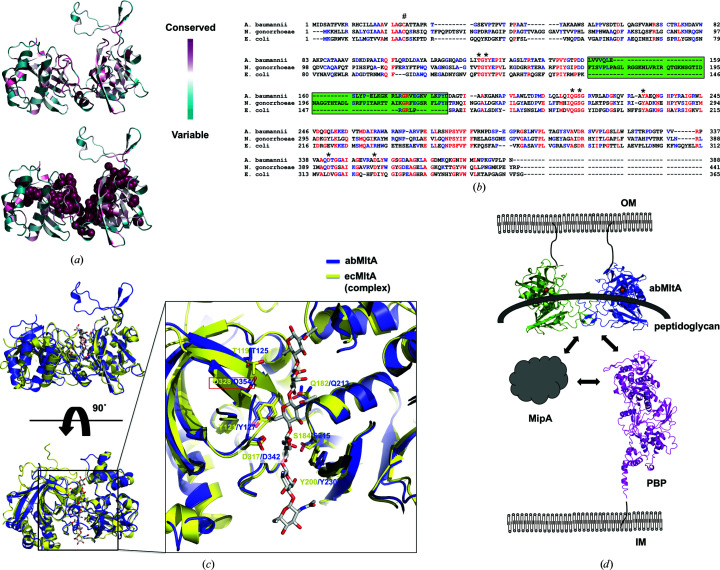
The tentative working mode of dimeric abMltA on the bacterial outer membrane. (*a*) Cartoon representation of abMltA colored according to the degree of amino-acid sequence conservation as analyzed by the *ConSurf* server. The side chains of completely conserved residues are shown with a ball-shaped model (lower panel) for better visualization. (*b*) Sequence alignment of MltA between different species. Mostly conserved and partially conserved residues are colored red and blue, respectively. The positions of the extra domain are highlighted by a green box. * indicates conserved residues that are involved in substrate binding. # indicates the conserved cysteine residue at the N-terminus of MltA, which is modified by lipid attachment for membrane anchoring. (*c*) Structural superposition of abMltA with the ecMltA–substrate complex. Seven residues that are involved in substrate recognition are labeled. The most critical residue directly involved in the cleavage of the substrate, Asp328 in ecMltA and Asp354 in abMltA, is highlighted using a red box. (*d*) Tentative working model of dimeric abMltA on the bacterial outer membrane. Red balls indicate the location of Asp354 (Asp328 in ecMltA), which is the most critical residue for the activity of MltA. OM and IM indicate the outer and inner membranes, respectively.

Analysis of the evolutionarily conserved amino-acid positions in abMltA based on the phylogenetic relations between homologous sequences using the *ConSurf* server indicated that the most conserved residues were located at the interface formed between domain 1 and domain 2, which possibly represents the substrate-binding pocket of abMltA [Fig. 4 ▸(*a*)]. Sequence alignment also showed that the previously identified amino-acid residues involved in substrate binding in the ecMltA system (including Thr119, Tyr121, Gln182, Ser184, Tyr200, Asp317 and Asp328) were completely conserved throughout different species, indicating that the strategies for recognition of the substrate in the active site might be very similar among MltA orthologs [Fig. 4 ▸(*b*)]. To analyze the substrate binding of abMltA, the structure of abMltA was superimposed with that of ecMltA complexed with a substrate analog. This structural comparison study showed that the locations of seven residues, Thr119, Tyr121, Gln182, Ser184, Tyr200, Asp317 and Asp328, involved in substrate binding in the ecMltA system were structurally conserved as Thr125, Tyr127, Gln213, Ser215, Tyr230, Asp342 and Asp354, respectively, in abMltA [Fig. 4 ▸(*c*)]. This confirmed that the seven conserved residues in abMltA might be used for the recognition of substrate via a strategy similar to that of the substrate recognition performed by the ecMltA system. Since the cysteine residue in the N-terminal region, which is a well known lipid-modification residue involved in membrane anchoring, was completely conserved across all species, the site of action at the membrane of MltA will be common [Fig. 4 ▸(*b*)]. The presence and the sequence of the extra domain vary among different species. ecMltA does not exhibit the extra domain, while the sequence and the structure of the extra domain of ngMltA were different from those of abMltA [Figs. 3 ▸(*i*) and 4 ▸(*b*)]. This indicated that the extra domain-mediated dimerization of MltA might be specific to abMltA.

Based on the results of previous studies and those observed in the current structural study, we conclude that MltA anchors on the outer membrane of the bacterial cell via lipid-modified cysteine residues at the N-terminus. Membrane-bound MltA recognizes the peptidoglycan using a deep substrate-binding groove, which is formed by seven highly conserved amino-acid residues and is involved in the peptidoglycan-recycling process with MipA and PBPs [Fig. 4 ▸(*d*)]. Although MltA functions in a monomeric form, as determined in a previous study performed using the representative Gram-negative bacterium *E. coli* (Powell *et al.*, 2006 ▸; van Straaten *et al.*, 2005 ▸), abMltA forms dimers in solution via an extra domain, indicating that MltA can function in both monomeric and dimeric states, the stoichiometric variation of which is dependent on the species [Fig. 4 ▸(*d*)].

## Supplementary Material

PDB reference: MltA, 7esj

## Figures and Tables

**Table 1:** Data-collection and refinement statistics Values in parentheses are for the outermost resolution shell.

Data collection	
Space group	*C*2
*a*, *b*, *c* (Å)	97.73, 67.44, 142.37
α, β, γ (°)	90, 98.87, 90
Resolution range (Å)	29.26–2.03
Total reflections	275146
Unique reflections	11895
Multiplicity	12.7 (11.33)
Completeness (%)	99.87 (99.83)
Mean *I*/σ(*I*)	11.5 (1.5)
*R*_merge_ †	0.090 (1.359)
Wilson *B* factor (Å)	43.55
Refinement	
Resolution range (Å)	29.26–2.06
Reflections	56714
*R*_work_ (%)	21.77
*R*_free_ (%)	26.40
No. of molecules in the asymmetric unit	2
No. of non-H atoms	
Total	5479
Macromolecules	5250
Solvent	229
Average *B*-factor values (Å)	
Macromolecules	35
Solvent	28
Ramachandran plot	
Favored (%)	96.37
Allowed (%)	3.63
Outliers (%)	0
Rotamer outliers (%)	0
Clashscore	5.71
R.m.s.d., bond lengths (Å)	0.008
R.m.s.d., angles (°)	0.929

† *R*_merge_ = \textstyle \sum_{hkl}\sum_{i}|I_{i}(hkl)- \langle I(hkl)\rangle|/\textstyle \sum_{hkl}\sum_{i}I_{i}(hkl), where *I*_*i*_(*hkl*) is the *i*th observed intensity of reflection *hkl* and 〈*I*(*hkl*)〉 is the average intensity obtained from multiple measurements.

**Table 2:** Structural similarity search using *DALI* (Holm & Sander, 1995 ▸)

Protein (PDB code)	*Z*-score	R.m.s.d. (Å)	Identity (%)	Reference
MltA from *N. gonorrhoeae* (2g6g)	37.2	3.2	35	Powell *et al.* (2006 ▸)
LtgG from *B. pseudomallei* (6qk4)	31.2	5.6	39	Jenkins *et al.* (2019 ▸)
MltA from *E. coli*, complex with chitohexaose (2pi8)	30.1	2.7	27	van Straaten *et al.* (2007 ▸)
MltA from *E. coli* (2pic)	18.4	8.8	27	van Straaten *et al.* (2005 ▸)
